# The Role of Organized Activities in Supporting Youth Social Capital Development: A Qualitative Meta-Synthesis

**DOI:** 10.1007/s40894-024-00235-1

**Published:** 2024-05-09

**Authors:** Ashley A. Boat, Heather Poparad, Miray D. Seward, Peter C. Scales, Amy K. Syvertsen

**Affiliations:** 1 Search Institute, 3001 Broadway Street NE #310, Minneapolis, MN 55413 USA; 2 American Institutes for Research, Arlington, VA USA

**Keywords:** Social capital, Organized activities, Adolescence, Thematic analysis, Meta-synthesis

## Abstract

Social capital provides young people with a web of supportive relationships that can be leveraged in pursuit of education, career, and life goals. Organized activities, an umbrella term for extracurricular activities, after-school programs, and youth development programs, are important developmental contexts for building social capital. The purpose of this study was to illuminate the developmental pathway through which social capital development occurs in organized activities. A qualitative meta-synthesis was conducted using 33 articles that met inclusion criteria across five databases (e.g., ERIC, PsycINFO) between June 2022 and May 2023. Thematic analysis was used to identify malleable organized activity features that act as levers for social capital promotion. Seven thematically aligned features were identified, including (1) organizational partnerships, (2) organizational supporting structures, (3) relationally strong climate, (4) staff mindsets and skills, (5) youth mindsets and skills, (6) increased social capital opportunities, and (7) increased social capital activation. These seven themes were used to construct an empirically-grounded model that posits a process through which organized activities support youth social capital development. Implications for intentionally strengthening organized activities’ capacity to support youth social capital are discussed.

## Introduction

Social capital is an important asset that adolescents and emerging adults can leverage while pursuing life course milestones including postsecondary education and employment. Social capital has been positively linked to progress towards education and career goals (Boat, et al., 2022a), increased support for securing a job (Brown et al., 2016), and career advancement including higher compensation levels (Ghosh & Reio, 2013; McDonald, 2015). And, it may be an especially valuable asset among students from underrepresented groups in higher education, including first-generation college students, students from low-income backgrounds, and students of color, who often experience systemic and institutional barriers that contribute to exclusion and inequitable academic outcomes (Mishra, 2020). Because social capital is a malleable asset that all youth possess to some degree and can utilize to reach their full potential, it is important to identify ways in which this critical resource can be intentionally strengthened. The current study defines social capital as the resources that arise out of a web of developmental relationships that young people can access and mobilize to improve their lives and achieve their goals (Scales et al., 2020), and examines how organized activities can support social capital development as youth pursue their education and career goals.

Research has largely focused on examining the contribution of family and school environments to youth social capital (Ryan, 2017; Stephan, 2013), and less emphasis on how organized activities that occur beyond the home and regular school day can be leveraged as forums for social capital development (the literature on school-business partnerships targeting youth from marginalized backgrounds has long been an exception, e.g., Scales et al., 2005). Organized activities are defined as structured and supervised activities outside the school curriculum that a group of young people engage in (Bohnert et al., 2010). This may include school-based extracurricular activities such as sports and clubs, as well as out-of-school time programming such as faith-based, youth, and workforce development programs. Plentiful research has shown that positive relationships provided through these activities support youth in pursuing their education and career aspirations (National Research Council, 2002; Vandell et al., 2015), but the literature has been less clear about exactly *how* structures and relationships in these contexts promote social capital. To address this gap, the current qualitative synthesis identifies and summarizes how organized activities intentionally support young people in strengthening and leveraging their existing social capital as they pursue their goals.

### Social Capital Theory

Social capital is a diffuse concept that has been reinvented multiple times over the last 40 years by a myriad of disciplines (e.g., economics, sociology, political science), which has resulted in multiple theoretical frameworks and definitions. While diverse, the majority of these definitions emphasize the value of resources that emerge via social relationships (e.g., Coleman, 1988; Freeland-Fisher, 2019; McPherson et al., 2014). Many of these foundational frameworks illustrate how individuals with more power and who have access to the ‘right’ kinds of capital benefit (Lehmann, 2019). For example, some scholars have noted that education and employment systems often value forms of capital (e.g., volunteering, internships) that favor youth from more privileged backgrounds (e.g., youth from higher-income families and/or communities who can do volunteer work without pay, or have transportation to get places easily; Moreau & Leathwood, 2006). Scholars have also critiqued these more traditional conceptualizations of social capital noting that these earlier descriptions of social capital are, “overly deterministic and tend to disregard the relevance of youth agency…” while also undervaluing the assets within young people’s communities of origin and suggesting that youth of color experience a “lack of social capital” (Akom et al., 2008, p. 2).

But there also have been examples over the last 20 years of more expanded definitions of youth social capital that draw from the fields of positive youth development, social justice, community organizing, and youth activism. The Community Cultural Wealth framework, for example, underscores various assets and resources utilized by communities of color while navigating social systems (Yosso, 2005). Similarly, critical social capital places a greater emphasis on relationships that engender community change while also centering how racial identity can serve as a resource for youth (Ginwright, 2007). These conceptualizations of social capital acknowledge structural constraints while also viewing youth as agents in facilitating change.

### The Inequitable Distribution of Social Capital

While social capital can provide a pathway for upward mobility (a goal that may be defined differently and considered more or less important across differing cultural groups), disparities in social capital among youth exist and contribute to unequal opportunities that further perpetuate inequities in postsecondary outcomes and career success (Timpe & Lunkenheimer, 2015). Young people of color and/or youth from lower-income communities face social and structural oppressions including disinvestment, poverty, and discrimination, all of which can constrain social capital development (Carpiano, 2006). There also are different forms of social capital, namely bonding, bridging, and linking, and each has its own potential to either thwart and/or advance the life trajectories of young people, i.e., either perpetuate or overcome the inequitable distribution of social capital (Scales et al., 2020).

Bonding social capital, which Coleman (1988) made central to his foundational and frequently-critiqued work in defining social capital, refers to connections between groups of individuals who share a group affiliation (e.g., culture, education, social class). This type of social capital can strengthen relationships between individuals who are already demographically similar (e.g., family members, close friends), and as a result can also serve as a basis for exclusion (Grootaert et al., 2004). For example, socially advantaged families may use and strengthen bonding social capital within their networks to amass educational opportunities for their own children (Murray et al., 2020), further exacerbating educational inequality for those without that same advantage (Calarco, 2018). Conversely, bridging and linking social capital both refer to connections across differing groups, with linking social capital more explicitly referring to connections made across power and status differences (Grootaert et al., 2004). These forms of social capital may have greater potential to influence equitable outcomes by enabling young people to access resources and information outside of their immediate social networks. Studies, for instance, show how staff, educators, and mentors may serve as ‘institutional agents’ (Stanton-Salazar, 2011), as well as how community-based organizations can serve as brokers (Dill & Ozer, 2019) by connecting youth from marginalized communities with pathways for opportunity that may not otherwise be available (e.g., internships, professional networking skills).

Due to increased access to individuals and institutions with power and/or prestige, youth from privileged backgrounds may have more bridging and linking forms of social capital (Bridwell-Mitchell, 2017). Therefore, these forms of social capital can heighten existing power dynamics, neglect systemic changes that are needed to yield more equitable education and economic opportunities for all youth, and ignore the roles that youth activism and collective efficacy can play in changing systems to be more equitable (Akom et al., 2008). Past theoretical work illustrates how young people from marginalized backgrounds often possess and commonly use forms of capital (i.e., aspirational, linguistic, familial, social, navigational, and resistant) that typically go unrecognized, unmeasured, and/or undervalued by dominant groups and institutions (Rabinowitz et al., 2020; Yosso, 2005). For example, one study found that when Hispanic/Latina/o/x students’ schools failed to provide them with information about postsecondary education opportunities, students tapped into the cultural wealth of their community to access information in other ways (Liou et al., 2009). Other studies have highlighted ways that youth of color have used their community cultural wealth to navigate inequitable systems to access higher education information (Welton & Martinez, 2014), resist racist policies and practices within institutions in order to reach postsecondary aspirations (O’Shea, 2016), and utilized influential support from family members while working towards educational goals (Gonzales, 2012). Therefore, understanding how organized activities can leverage young people’s wealth of assets by fostering social capital development may be particularly important for youth who may experience marginalization in school or other spaces that undervalue these forms of capital.

### Organized Activities and Youth Social Capital Development

Organized activities may be uniquely positioned to help youth recognize and activate forms of social capital that they already possess, as well as intentionally support young people in strengthening and leveraging their social capital while pursuing life goals. Prior research suggests that participation in organized activities is positively linked with greater academic motivation and achievement (Hughes et al., 2016), greater likelihood of employment (Heath & Thornock, 2022), and increased civic engagement (Zarrett et al., 2021). Additionally, organized activities are known to serve as important contexts for positive youth development and growth, where young people have opportunities to build skills (e.g., relationship-building, social skills), overcome challenges (e.g., peer pressure, academic struggles), and foster prosocial relationships (Scales, 2017; Vandell et al., 2015).

While participation in organized activities is not in itself social capital, they may provide the conditions in which young people can build and strengthen social capital. For example, participation in organized activities creates opportunities for youth to develop close relationships with peers and supportive adults both in and outside of the activity, which can create new avenues to access additional resources (e.g., knowledge, new connections; Vandell et al., 2015). However, organized activities can fall short in providing youth with multiple positive relationships and opportunities to exert agency, pursue deep interests, and enhance skills (Scales, 2017). In the worst cases, some organized activities paint a deficit-based portrait of youth, especially youth from historically marginalized backgrounds, as being in need of “saving” (Baldridge, 2014). Therefore, it is important to identify the core components of high-quality organized activities that intentionally support the social capital development of *all* young people from a strengths-based perspective.

National studies conducted by the Afterschool Alliance (2020) point to alarming (and growing) disparities in young people’s access to organized activities in the afterschool hours. Their most recent 2020 study found that 36% of youth in grades 9 to 12 would participate in an afterschool program if one were available. This unmet need is even more pronounced among low-income Black and Hispanic/Latina/o/x children. Even when programs do exist parents cite cost (57%) and lack of transportation (62%) as barriers to participation. Unequal access to organized activities translates into unequal access to critical developmental contexts and opportunities shown to promote a range of social-emotional, educational, civic, and psychological benefits, and—as posited here—social capital development.

## The Current Review

The current review uses qualitative meta-synthesis research methods to synthesize existing qualitative studies in order to develop a theoretical understanding of how organized activities support young people’s social capital development while they pursue education and/or career goals. In this work, we aim to identify the malleable features of organized activities that programs can feasibly influence to intentionally strengthen social capital for *all* young people, including youth from historically minoritized and marginalized communities. The growing body of qualitative research focused on youth social capital development has matured to a point where we believe that a synthesis of this literature from both youth and staff perspectives would help the field better understand the ways in which organized activities intentionally promote youth social capital. Therefore, the current study aimed to identify the elements within organized activities that intentionally support young people’s social capital development as they pursue education and career goals.

## Methods

The qualitative meta-synthesis followed a six-step method (Lachal et al., 2017): (1) conduct a literature search and define the inclusion/exclusion criteria; (2) use selection criteria to screen studies; (3) conduct a critical appraisal of included studies; (4) extract and present data; (5) conduct a thematic analysis; and (6) report the synthesis. Steps 1–3 are discussed below along with a description of our thematic analysis methodology. Steps 4–6 are presented in the Results.

### Literature Search and Inclusion/Exclusion Criteria

Studies included in the review were located through a multi-pronged search process conducted between June 2022—May 2023. First, a literature search was conducted for journal articles written or published between the years 2000 and 2023, utilizing five databases: PsycINFO, ERIC, Sociological Abstracts, Web of Science, and Academic Search Premier. In these database searches, keywords were specified to capture organized activities that focused on building young people’s social capital development while they work towards education and/or career goals.

The keywords used to capture organized activities included: OST, out-of-school time, extracurricular, organized activities, organized program, work-based learning, workforce development, youth development program, youth mentoring, community-based organization, and after school program. Keywords for social capital included: social capital, social network, social support, webs of support, relational support, peer support, and support network. Keywords for education and/or career outcomes included: education aspirations, educational attainment, college enrollment, academic motivation, academic performance, academic achievement, work readiness, employment, career interests, and occupational identity. Boolean search operators were utilized to connect keywords across these three domains. We chose not to apply qualitative filters (i.e., search engine filters that specify qualitative studies only) in order to capture all the possible relevant articles.

Studies needed to meet the following criteria to be eligible for final review: (1) used a qualitative research design such as semi-structured interviews or focus groups; (2) examined a specific organized activity (as previously defined); (3) examined young people’s social capital (which needed to include discussion of resources that arise from relationships); (4) was an original empirical article published between 2000 and 2023; (5) was written in English; (6) derived from data collected in the United States; and (7) focused on adolescents and/or emerging adults (ages 14–24). If the organized activity’s target audience extended outside of the 14–24 age range and was inclusive of this range, the study was still eligible for inclusion.

Studies were excluded from the review if they met any of the following criteria: (1) used only a quantitative research design; (2) occurred *only* outside of the United States (studies that include multiple countries were eligible if an organized activity in the United States was also included); (3) written in a language other than English; (4) published before 2000 (excepting studies that would reasonably be considered foundational or classic pieces of research, that are judged by the authors to still be relevant. However, none of these types of studies emerged); (5) included only a one-off and/or standalone activity such as a 1-day job fair; and/or, (6) exclusively focused on online social networks or natural mentoring relationships that are not facilitated via an organized activity.

### Screen Studies

**Fig. 1:**
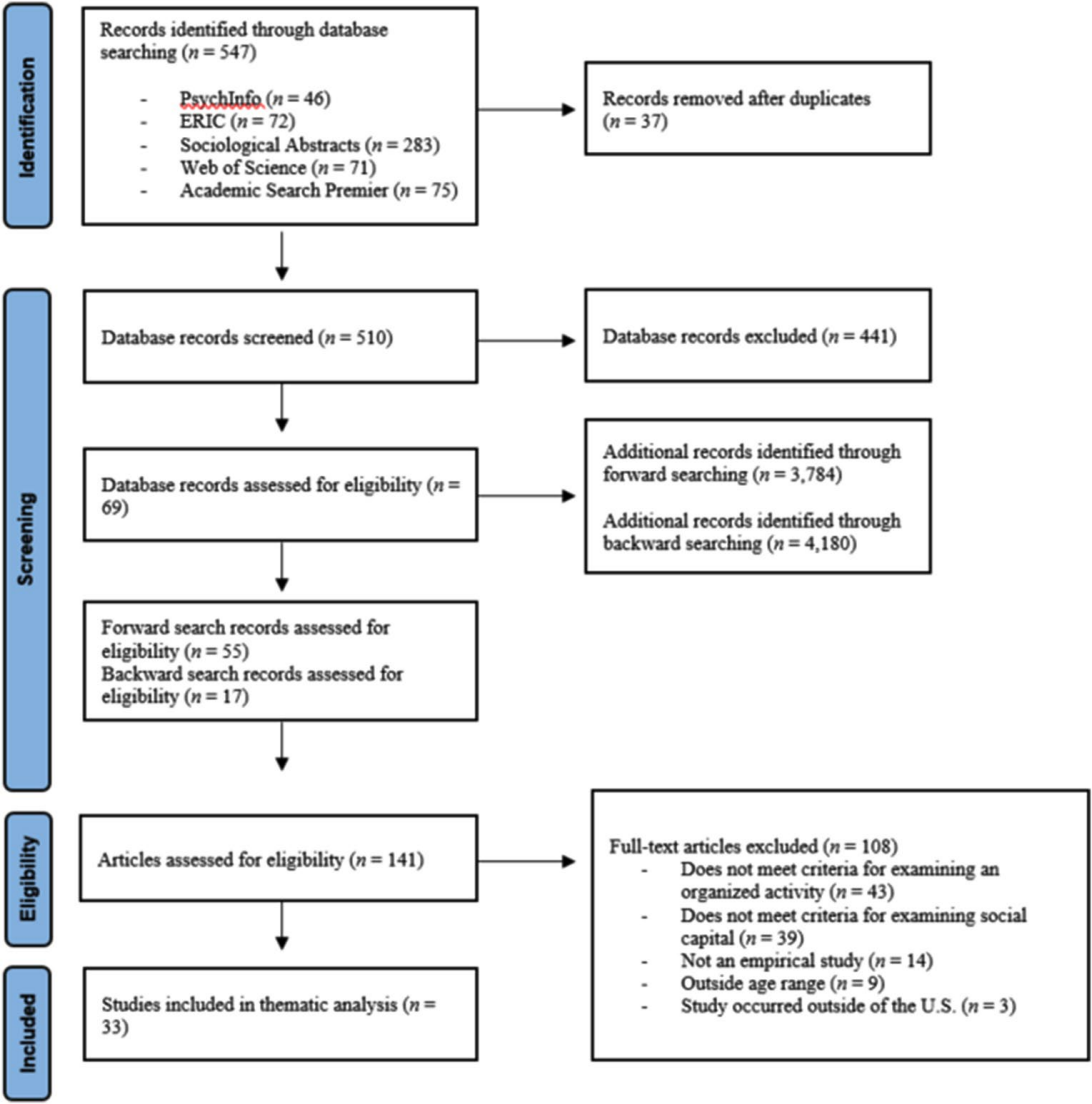
PRISMA flow chart for social capital in organized activities meta-synthesis

The initial literature search utilizing the five databases yielded 547 records (see Fig. 1). Titles and abstracts from the database search were screened by the first or second author to identify an initial set of 69 unique articles for further review. Subsequently, the first and second author completed a forward search by using the “cited by” function in Google Scholar for each of the 69 unique articles that emerged from the original database search. This forward search yielded 3,784 total records. These authors examined the title and abstracts of these additional forward search records to determine if any of these articles warranted additional review. An additional 55 unique articles were identified from the forward search process. Finally, a backward search was conducted by the first author by searching through the reference list of articles that were selected for further review following both the database and forward search. This resulted in 17 additional articles for further review. Taken together, this search process yielded a total of 141 records that warranted closer review.

To ensure interrater reliability in the inclusion/exclusion of potential articles, the first and second author both independently read 50 of the 141 articles in full and recorded which of these studies met inclusion/exclusion criteria (interrater reliability = 94% agreement). The first and second author then read the title, abstract, and Methods section of the remaining 91 articles to determine whether they met inclusion/exclusion criteria. Following this subsequent review, 94 articles were selected for full-text review. Both the first and second author read these articles in their entirety and any disagreements and/or discrepancies about which articles warranted inclusion in the final review were discussed until consensus was reached. During this review process, all types of research methods (i.e., qualitative, quantitative, and mixed methods) were accepted. However, of the few quantitative studies identified through the review process, none provided entirely original or novel information—beyond what was found in the qualitative studies—on the core elements of organized activities that support young people’s social capital development. This process resulted in a final set of 33 articles that were identified for inclusion in the current synthesis.

### Critical Appraisal

In addition to screening each article for inclusion, we also used the Critical Appraisal Skills Programme (CASP; 2018) checklist, a 10-item quality assessment tool, to appraise the quality of studies included in the synthesis. The CASP was used because it provides a systematic process for identifying the strengths and weaknesses of qualitative research studies and is considered to be user-friendly (Long et al., 2020). The CASP does not have a scoring system, but instead asks users to indicate whether studies address each of the 10 items using the following response options: *Yes, No, Can’t Tell*. Based on recommendations from past researchers and our own use of the tool, we decided to add a fourth response option: *Somewhat* (Long et al., 2020). The first three authors reviewed each of the 33 studies and based on the CASP criteria they organized the studies into three quality categories: low (scored yes or somewhat on 4 or fewer CASP items), medium (scored yes or somewhat on 5–7 CASP items), and high (reported yes or somewhat on 8 or more CASP items). Of the 33 studies, one was categorized as low quality, 13 were categorized as medium, and 20 were categorized as high. The sole study categorized as “low” was examined a second time in order to determine whether the reasons for this classification were due primarily to missing information (i.e., *Can’t tell*) or whether the overall methodology was poor. It was determined that the score was primarily due to missing information. Based on this review, we made the decision to not exclude any studies from our synthesis. While the authors of some of the reviewed studies may have failed to describe their methods in sufficient detail for us to determine whether criteria had been met (*Can’t tell*), we did not believe this lack of reporting necessarily translated into a poorly conducted study and felt that all 33 studies contributed to our understanding of our research question.

### Thematic Analysis

The 33 articles that were retained were coded in order to identify themes. Three coders (1st, 2nd, and 3rd author) worked together to code the articles in Dedoose (2021). Utilizing thematic analysis techniques (Braun & Clarke, 2006), the three coders first familiarized themselves with the data by reading through all of the articles. While reviewing, coders paid particular attention to any text relating to social capital in the Results and Discussion sections. This included participant quotes and/or primary author interpretation. A combined deductive and inductive approach was used to identify codes. The coders made note of potential codes that included elements of organized activities that supported youth social capital development, as well as preliminary codes that aligned with the authors past theory-building and social capital research (Boat et al., 2022b; Scales et al., 2020). Yet, authors remained open and allowed for the identification of new codes and themes throughout the coding process. The three coders used this initial set of codes to independently code the same article before meeting to discuss and refine the codebook. From there, each coder was assigned 11 articles to code independently. Coders met weekly to discuss the utility of the existing codes and potential modifications, emergent themes, and to reach consensus on how to code data that was unclear. Codes were then reviewed and organized into broader themes that speak to the mechanisms by which organized activities support youth social capital development.

## Results

### Description of the Studies

Table 1 provides a description of the 33 studies retained for coding. All of the studies were purely qualitative studies with the exception of two studies that used mixed methods (Bempechat et al., 2014; Schwartz et al., 2018). Articles examined a range of different types of organized activities—including afterschool programs, work-based learning, STEM enrichment, mentoring programs, sports, and clubs, among others. All of these organized activities were designed for youth in high school and/or college, with many serving youth of color. Table 1 provides more information on who the organized activities served and were designed for.

**Table 1:** Description of articles included in the final review

First author	Year	Type of organized activity	Age range of youth participants	Racial/ethnic identity of youth participants	Themes present
Barrett	2010	Black church	All ages	Intentionally serves the African American/Black community.	OSS; RSC; SSM; YSM; SCO
Bempechat	2014	Work-based learning	High school aged	Study participants identified as 40% Black/African American, 40% Hispanic/Latina/o/x, 5% White, 3% Asian/Asian-American, and 12% Other.	OSS; RSC; SSM; YSM; SCO
Cayleff	2011	School-based club	14–18	Study participants identified as White *(n* = 2), African American (*n* = 1), Biracial (*n* = 1), Hispanic/ Latina/o/x *(n* = 2), Vietnamese (*n* = 1), and European (*n* = 1).	OP; RSC; SSM; YSM; SCO; SCA
Ching	2016	Afterschool program	High school aged	States that the program is committed to reaching youth from low-income and non-dominant communities. Racial/ethnic background for study participants is not provided.	OP; RSC; SSM; YSM; SCO; SCA
Cox	2017	Preparatory program for elite boarding schools	14–15	Intentionally serves Black and Latina/o/x students from low- or moderate-income families. Percentages of racial/ethnic identity not provided for study participants.	OP; OSS; RSC; SSM; YSM; SCO; SCA
Detgen	2021	College and career readiness program	14–18	57% of study participants from the United States (racial/ethnic identity not provided). 43% of the study participants were from Kenya, Scotland, South Africa, Italy, Romania, Spain, Panama, or Columbia.	OP; OSS; RSC; SSM; YSM; SCO; SCA
Dill	2019	Community- based organization	12–20	Study participants identified as 80% African American, 20% Latina/o/x.	OP; OSS; RSC; SSM; SCO; SCA
Erbstein	2013	Community- based organization	12–18	Percentages are not provided but indicated that study participants were predominantly Hispanic/Latina/o/x.	OSS; SSM; YSM; SCO
Garcia	2021	Alliance-based STEM enrichment program	Undergraduate students	Study participants identified as Black (*n* = 11), Latina/o/x (*n* = 10), Black/ Latina/o/x (*n* = 4), Latina/o/x /White (*n* = 3), and Black/White (*n* = 2).	OP; OSS; RSC; SSM; YSM; SCO
Ginwright	2007	Community- based organization	6–18	100% of study participants identified as Black/African American.	OP; OSS; RSC; SSM; YSM; SCO; SCA
Habig	2021	STEM enrichment program	5th − 12th grade	Study participants identified as Latina/o/x (*n* = 1), Black/African American (*n* = 1), and Asian/Pacific Islander (*n* = 1).	OSS; RSC; SCO; SCA
Hargrave	2015	STEM after-school program	8th − 12th grade	100% of study participants identified as Black/African American.	OP; OSS; RSC; SSM; YSM; SCO
Harris	2015	Community- based and school-based programs	7th - college	100% of study participants identified as Hispanic/Latina/o/x.	OP; OSS; RSC; SSM; YSM; SCO; SCA
Hernandez- Gantes	2018	IT career readiness program	High school aged	Program serves students who identify as 54% as White; 25% as Hispanic/ Latina/o/x, 13% as African American, 2% as Multiracial, 5% as Asian, 0.5% as American Indian, 0.5% as Pacific Islander.	OP; OSS; RSC; SCO
Hos	2019	Newcomer program	13–18	Intentionally serves immigrants. Percentages of study participants are not provided but indicate that youth are predominantly Burmese/Karenni refugees from Thailand.	OP; OSS; RSC; SSM; YSM; SCO; SCA
Jarrett	2005	Rural school-based, urban arts, and urban civic program	High school aged	One program predominantly serves youth who identify as White. Two programs predominantly serve racially and ethnically diverse youth (percentages not provided).	OP; OSS; RSC; SSM; YSM; SCO; SCA
Kniess	2020	Life skills development program	18–23	Study participants identified as 87.5% African American and 12.5% Mexican American	OSS; RSC; SCO; SCA
Koch	2019	Afterschool STEM program	10th grade	100% of study participants identified as Latina.	YSM; SCO
Lane	2020	STEM summer program	15–23	100% of study participants identified as Black/African American	OSS; RSC; SSM; YSM; SCO;
Miller	2011	Entrepreneurship program	Undergraduate students	Study participants identified as White (*n* = 17), African American (*n* = 11), and Latina/o/x (*n* = 2).	OP; OSS; RSC; SSM; YSM; SCO;
Murillo	2017	School-based internship program	12th grade	Racial/ethnic identity information was not provided.	OSS; RSC; SSM; YSM; SCO; SCA
Museus	2010	Targeted support programs	Undergraduate students	Study participants identified as Asian American (*n* = 9), Black/African American (*n* = 9), and Latina/o/x (*n* = 13).	OP; OSS; RSC; SSM; YSM; SCO
Nolen	2020	Student-led clubs	Undergraduate and graduate students	Intentionally serves Latina/o/x/Hispanic students. Study participant racial/ethnic identity data was not provided.	OP; RSC; SCO
Powell	2017	Workforce development program	18–24	100% of study participants identified as African American.	OP; RSC; SSM; YSM; SCO
Ramirez	2021	Community- based organization	High school to college aged	Study participants identified as 50% Black/African American and 50% Latina/o/x.	OP; OSS; RSC; SSM; YSM; SCO; SCA
Richardson	2012	Sports	7th- 9th grade	100% of study participants identified as African American/Black.	OP; OSS; RSC; SSM; YSM; SCO; SCA
Sanchez	2022	Mentoring program	High school and college aged	100% of study participants identified as Hispanic/Latina/o/x.	OP; OSS; RSC; SSM; YSM; SCO; SCA
Schwartz	2016	Skill-building program	17–18	Study participants identified as 92% Haitian and 8% Cape-Verdean.	RSC; YSM; SCO; SCA
Schwartz	2018	Skill-building program	College students	Study participants identified as 32.5% Asian, 23.9%, Hispanic/Latina/o/x, 19% Black, 11.7% White, 8% multiracial, 5% Other.	YSM; SCO; SCA
Sullivan	2010	Leadership programs, career training programs	13–21	Study participants identified as 33.8% Hispanic, 29.6% European American, 26.8% African American, 7.0% Biracial, and 2.8% Asian	OP; OSS; SSM; YSM; SCO
Tichavakunda	2019	Afterschool program	14–19	100% of study participants identified as Hispanic/Latina/o/x.	OP; RSC; SSM; YSM; SCO; SCA
Wong	2008	Community- based organization	11–18	100% of study participants identified as Chinese American.	OP; OSS; RSC; SSM; SCO
Woodland	2009	Afterschool program	15–23	100% of study participants identified as Black/African American.	OSS; RSC; SSM; YSM; SCO; SCA

*Note* OP = Organizational Partnerships; OSS = Organizational Supporting Structures; RSC = Relationally Strong Climate; SSM = Staff Skills & Mindsets; YSM = Youth Skills & Mindsets; SCO = Social Capital Opportunity; SCA = Social Capital Activation

### Thematic Analysis

**Fig. 2:**
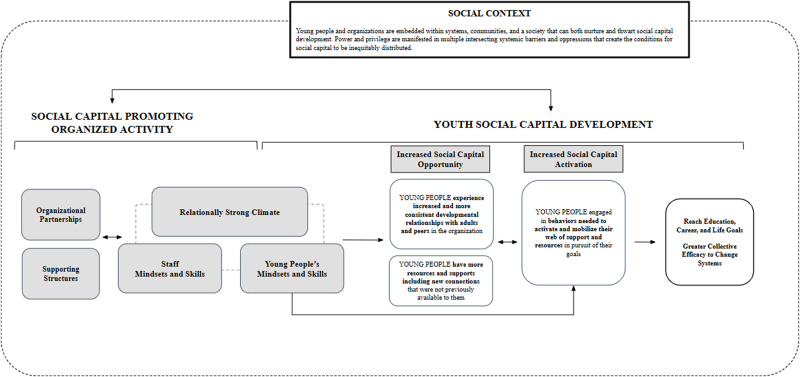
Proposed model for how organized activities support young people’s social capital development

From the data, seven thematically aligned strategies were identified for how organized activities support youth social capital development, including (1) organizational partnerships, (2) organizational supporting structures, (3) relationally strong climate, (4) staff mindsets and skills, (5) youth mindsets and skills, (6) increased social capital opportunities, and (7) increased social capital activation. Because these seven identified themes are interconnected and suggest a potential pathway through which key elements of organized activities may be leveraged to intentionally build social capital, we constructed an empirically-grounded model that organizes these themes and posits a process through which organized activities support youth social capital development (see Fig. 2). The themes of organizational partnerships, organizational supporting structures, relationally strong climate, and staff and youth mindsets and skills feed into creating a social capital promoting organized activity. The themes of increased social capital opportunity and activation illustrate how the elements of a social capital promoting organized activity support youth social capital development as youth pursue life goals. Each of the themes and their corresponding sub-themes are described below with supporting quotes from primary data.

### Social Capital Promoting Organized Activity

While primarily interested in the ways that organized activities support individual-level social capital, the synthesis showed the important role and impact of social capital at the organization level. In addition to organizational partnerships, findings underscore several supporting structures that were helpful for cultivating a relationally strong climate that prioritized youth social capital development. This relationally strong climate was characterized as a safe and culturally responsive space where youth feel a strong sense of community and belonging, and are provided opportunities to explore their sparks; i.e., the interests and passions that give them joy, energy, and purpose (Scales et al., 2023). Findings also identified staff and youth skills and mindsets that likely nurture this climate.

#### Organizational Partnerships

The meta-synthesis showcased how social capital does not just exist between people, it also exists at the organization level whereby administrators and directors of organized activities form partnerships with other community-based organizations that allow the youth they serve to access additional value-add resources. For example, a career readiness program built partnerships with a local network of information technology (IT) employers to give youth first-hand exposure to the kind of careers they could pursue in the IT field (Hernandez-Gantes et al., 2018). This type of relationship building often resulted in increased opportunities (e.g., internships, informational interviews) for youth and allowed them to imagine themselves in different kinds of careers. Another study of a STEM program serving African American/Black youth highlighted the resources that emerged from the program’s partnerships with community businesses: “in addition to financial support, STEM businesses provided students, teachers, and parents with access to professional scientists and engineers and cutting-edge science research facilities” (Hargrave, 2015, p. 10). Some organized activities became well-respected institutions within their communities which further allowed their leaders to obtain more partnerships and positions that further increased access to opportunities. For example, the pastor of a community-based church joined the Board of Education in hopes of addressing inequalities in the local education system. The church also created community-wide opportunities including opening a franchised restaurant on the church campus to address community unemployment rates, while giving Black high school students opportunities to gain work experience and see an example of a successful Black man who owns his own business (Barrett, 2010).

#### Organizational Supporting Structures

The extant literature points to the structural supports organized activities rely on when building youth social capital development. These supporting structures include retention of high-quality staff, presence of a shared organizational mission and values, and hiring staff with similar identities and/or life experiences as the youth served.

##### High-Quality Staff Retention

In order to effectively meet the needs of young people and support their social capital development, it is essential that organized activities retain and support high-quality staff and volunteers (Hall et al., 2020). For example, a newcomer program (i.e., programs designed in K-12 settings to support large influxes of refugees and immigrants) that served immigrant and English learner students grappled with low retention of qualified educators, and had to staff classrooms with educators who did not have Teaching English to Speakers of Other Languages (TESOL) training, and who were certified in other subjects than the one they were assigned to teach (Hos et al., 2019). Conditions like these make it exceedingly difficult for organized activities to adequately meet young people’s needs. In contrast, when organized activities retain high-quality staff with effective training and experience it can lead to a profound impact for youth participants. For example, the newcomer program described a teacher, Mrs. Smith, who was particularly gifted at supporting student needs by explicitly drawing on students’ lived experiences and using effective modeling techniques (Hos et al., 2019). Organized activities with successful staff retention efforts are also better positioned to establish long-term youth-adult relationships and trust within the communities they serve. For example, one community-based program described how their “newest” adult staff member had been at the organization for over 8 years, which led to “long-term relationship building and created institutional memory” (Dill & Ozer, 2019, p. 1620). This organized activity was likely able to retain staff due to its strong presence in the community and hiring of staff that were both from and committed to supporting youth within the community.

##### Shared Organizational Mission and Values

Staff enhance their ability to collaborate in service of youth when they embody a set of shared values and a collective commitment toward achieving agreed-upon outcomes. A career readiness program illustrated this alignment among staff when a staff member observed that “everywhere we went, the message was the same: the commitment to work together as a community bridging and linking school- and work-based strategies was crucial in supporting and sustaining the promotion of career readiness for IT Academy students” (Hernandez-Gantes et al., 2018). Working together creates a ripple effect that influences relationships with community partners, and the relationships that those community partners develop with the program’s youth. In a high school STEM program with a university affiliate, “… the interactions with university staff and teachers was where program practices and dispositions were shaped to ensure consistency and fidelity between what was said and what was done” (Hargrave, 2015, p. 358). The interactions between teachers and university staff ultimately influenced the program’s rhetoric and programming, adults’ beliefs about youth potential, and messaging that youth received from staff and community partners. When staff are unified under a shared mission, a cohesive set of goals, values, and supports are amplified for youth.

##### Staff Share Similar Identities and/or Life Experiences as Youth

Employing staff who share similar identities (e.g., racial, cultural, gender) or life experiences as the youth served can promote a sense of belonging (Garcia et al., 2021; Wong, 2008). For example, a young person in an academic enrichment program reflected on her relationship with a Hispanic/Latina educator who earned a Doctorate in Education: “[She]… was so inspiring. Wow, I could be you. I look up to you. You look like me. You speak like me. We share a language… and it’s possible” (Habig et al., 2021, p. 519). When youth are able to identify with and see themselves reflected in the adults in their organized activities, it is easier to dream about pursuing similar futures for themselves. Shared identities and life experiences provide a common ground for adults to connect with, understand, and provide culturally relevant support to young people (Erbstein, 2013; Wong, 2008), which can yield more trusting youth-adult relationships (Garcia et al., 2021). For youth of color, in particular, identification with adult staff from similar backgrounds can support more than a sense of belonging, contributing also to the recognition that their culture is an asset for developing a network of aspirational role models with whom they can relate (Habig et al., 2021). As such, employing and hiring staff who share common identities and/or life experiences with youth is fertile ground for developing relationships with positive adults who motivate them to dream big and achieve their goals.

#### Relationally Strong Climate

The organized activities included in the meta-synthesis highlighted the characteristics of a strong relational climate. Organized activities that were experienced as safe and culturally responsive—where youth have a strong sense of community and belonging, and are given opportunities to explore their interests, passions, and sparks—were identified as ripe for cultivating youth social capital.

##### Create a Safe Environment

To create a relationship-rich climate, it is important for youth to feel physically and emotionally safe within the organized activity. One way this was achieved, for example, was through coaches creating “safezones,” within organized sports such as basketball and boxing leagues which provided *physically* safe spaces for Black youth who were living in neighborhoods with high levels of community violence (Richardson, 2012, p. 190). Coaches noted that these protected social spaces on and off the athletic field/court were used as a “safe haven and buffer from violence within the local community” (Richardson, 2012, p. 190). While this program highlighted the importance of physical safety, many of the other organized activities reviewed described how through participation in the organized activities, youth felt *emotionally* safe, which often resulted in them expressing their authentic selves. For example, an afterschool program employed “facilitators [who] were aware that the youth needed to feel that they had a space where they could express themselves and their identities and ideas, and were safe from stereotypes, belittlement, and intimidation” (Woodland et al., 2009 p. 243). Other organized activities created safe spaces for Hispanic/Latina/o/x youth by engaging in initiatives that allowed for the development of “confianza (mutual trust)” between youth and adults enabling youth to “fully embrace all aspects of who they are” (Harris & Kiyama, 2015 p. 194). Creating environments of physical and emotional safety fostered trust and enabled youth to build strong and more meaningful relationships while staying true to their values and beliefs.

##### Engage in Culturally Responsive Ways

A culturally-responsive climate was manifested through interactions with staff and culturally-grounded instruction and materials that were personally relevant to youth. For example, a community-based program described how its participants who identified as Chinese American commented on how the materials were culturally sensitive (e.g., materials written in multiple languages, resources for engaging families, bilingual and bicultural teachers and lessons), with one participant stating, “I could relate more here [referring to program] but not at school” due to the culturally-relevant materials (Wong, 2008, p. 190). Black youth in an afterschool program shared that it was the culturally-relevant discussions about topics such as history, popular culture, and their personal experiences that attracted them to the organized activity and encouraged further participation (Woodland et al., 2009). Several of the organized activities also described providing a space for youth of color to discuss systematic barriers and experiences of discrimination with staff and others. For instance, one mentoring program illustrates how near-peer mentors of color tried to prepare their mentees for uncomfortable encounters of discrimination and biases that they themselves had experienced (Cox, 2017). Organized activities were also sensitive to youths’ needs and context, which was particularly beneficial for supporting youth-adult relationship building across lines of difference (e.g., differences in race and/or socioeconomic status; Ramirez, 2021). Examples included bridging parent-school relationships in programs for Hispanic/Latina/o and Chinese American youth (Erbstein, 2013; Wong, 2008) and providing resources (e.g., access to education and job opportunities, financial support) to help overcome systemic barriers in programs for Thai refugee youth and Chinese American youth (Hos et al., 2019; Wong, 2008). It is important to note that in some of the organized activities, staff were able to be culturally responsive to youth because they themselves were from the same communities and/or had experienced the same life events (e.g., Cox, 2017); whereas in other organized activities, staff who did not share these same life experiences were educated through their interactions with young people (e.g., Ramirez, 2021) and/or through explicit training (e.g., Sanchez et al., 2022).

##### Build a Strong Sense of Community and Belonging

Organized activities also created a strong relational climate through fostering community and a sense of belonging. Across the organized activities examined, staff and youth often described their organized activities as a “second home,” a family, and/or a team (Bempechat et al., 2014; Powell et al., 2017; Tichavakunda, 2019). For example, staff in a 14-month program, called Launch, that prepares low-income students of color to attend elite boarding schools through mentorship and academic training referred to staff and participants as a family in stating, “… your fellow crew members are your ‘brothers’ and ‘sisters’ within the larger Launch ‘family’…” (Cox, 2017 p. 54). In some cases, youth joined organized activities to increase their feeling of belonging (e.g., Nolen et al., 2020). Organized activities often represented chosen communities, where youth found connections with peers who share similar interests or life experiences as their own. For example, a career readiness program described how it “celebrated their common linkages and bonds,” as all the young people shared a “communal identity” (Hernandez-Gantes et al., 2018, p. 196). The sense of belonging that was cultivated in organized activities engendered a supportive community that young people could be a part of and rely on.

##### Provide Opportunities to Explore Sparks

The space that organized activities create for youth to explore their sparks or deep personal interests (Scales et al., 2011) sets them apart from other contexts like schools where the curriculum is largely driven by educational standards. One afterschool program did this through field trips that gave youth opportunities to learn more about newfound interests, future possibilities, and other communities and places (Ching et al., 2016). Organized activities also allowed youth to explore a variety of different sparks that were tailored to their unique interests. For example, a staff member of an organized activity that focused on developing young entrepreneurs stated, “The most basic goal we have is to help them get in touch with what they’re about. Because everyone has their own unique set of personal gifts. Defining what these gifts are and what the needs of the world are and finding out where they overlap - that’s what we shoot for…” (Miller, 2011, p. 53). Youth may be more likely to make progress towards their life goals when opportunities and resources within organized activities are aligned with their sparks (Scales et al., 2011).

#### Staff Mindsets and Skills

Staff have specific mindsets and skills that nurture a strong relational climate. These include believing in youth assets and potential, and skills such as authentic relationship-building skills and brokering skills.

##### Staff Belief in Youth Assets and Potential

In organized activities, staff’s beliefs in the assets and potential of youth are sometimes in stark contrast to what youth experience from other adults in their schools and communities (e.g., Ginwright et al., 2007). In many organized activities, staff were described as acting in ways that affirmed young people’s aspirations (e.g., Lane & Id-Deen, 2020; Ramirez, 2021). At the same time, they continuously held high expectations for youth and balanced support with autonomy to meet those expectations (e.g., Murillo et al., 2017; Sanchez et al., 2022). For youth of color, this support inspired them to challenge deficit narratives by working to engender program or community changes that might facilitate their academic and professional success. Illustrating this experience, a Black youth participating in a leadership program shared, “They [staff] see me as an activist or something, and I’m not political like that. But when [the program director] lets me speak my mind to folks like the mayor and political people, it makes you want to live up to that image, you know” (Ginwright, 2007). A faith-based organization provides another example where the pastor created a community that held high expectations and belief in Black youths’ value by providing public recognition and celebration of academic success through its academic achievement days (Barrett, 2010). When staff demonstrate belief in young people’s potential, they empower youth to imagine other possible selves. Staff also recognized and valued the resources and support that youth, their families, and communities already possessed (e.g., encouragement, high expectations, being held accountable), and found ways to leverage these assets by building strong relationships with youth’s families and communities to further strengthen these existing relationships (Erbstein, 2013; Lane & Id-Deen, 2020).

##### Staff Authentic Relationship-Building Skills

Staff who built strong relationships with the youth they served did so by being intentional, equitable, and inclusive in their relationship-building efforts. Staff sometimes found themselves taking on roles beyond what was expected and written down in their job title in order to meet the needs of youth. For example, one sports coach stated, “I wore multiple hats in this setting: social scientist, coach, chaperone, chauffeur, and counselor” (Richardson, 2012, p. 188). Staff in a community-based program intentionally worked to “​​get to know youth’s family and teachers, they chaperone them [youth] to field trips, they act as interventionists for the youth at their schools and in their neighborhoods” (Dill & Ozer, 2018, p. 1620). In addition to meeting youth needs by playing multiple roles, staff who possessed the skills to establish trusting relationships were able to gain “… access to intimate life details and student knowledge,” which enhanced their ability to provide effective supports (Ramirez, 2021, p. 1075). This was especially valuable for youth from marginalized backgrounds, as youth were more apt to openly talk to staff about structural barriers that they were facing, enabling staff to advocate for youth and act as “social brokers’’ (Ramirez, 2021, p. 1075). Because staff had the skills to build these strong relationships with youth, they were better positioned to support young people’s academic, social-emotional, and professional needs (Lane & Id-Deen, 2020). In doing so, they could empower youth to believe in themselves and to approach postsecondary goals with the mindset that they deserve to be stretched to develop to their fullest potential.

##### Staff Brokering Skills

Cultivating trust with youth and providing them with the needed supports opened up opportunities for staff members to act as brokers of relationships and opportunities (e.g., Sanchez et al., 2022). In organizations’ attempts to leverage these youth-adult relationships to enhance youth’s social capital, staff were often positioned as institutional agents who assisted youth in navigating unfamiliar systems (e.g., Museus, 2010; Ramirez, 2021). Consequently, these staff members often served as brokers to access other connections, resources, and opportunities that youth could benefit from (e.g., Hos et al., 2019). Most frequently, staff brokered relationships and connected youth with resources that existed outside of the organized activity (e.g., postsecondary related services, admissions personnel, research opportunities; Museus, 2010; Ramirez, 2021; Sanchez, 2022). Often, this brokering entailed providing youth with the “hook-up” (Dill & Ozer, 2018, p. 1622), and drawing “on the program’s connections to identify potential relationships, and [vouching] for the youth to the community adults (putting their own reputations on the line)” (Jarrett et al., 2005, p. 53). By vouching for youth in organized activities, staff were able to help youth gain “access to educational opportunities that they otherwise would not have access to” (Garcia et al., 2021, p. 7).

#### Youth Mindsets and Skills

Youth also have specific mindsets and skills that contribute to a strong relational climate, and are also likely to contribute to enhanced ability to actively mobilize social capital as they work towards their life goals. These mindsets and skills include having a future-oriented mindset, commitment to paying-it-forward, self-efficacy to reach goals, and strong relationship-building skills.

##### Youth Future Orientation

Organized activities exposed youth to potential futures under the support of caring staff who helped them navigate their future goals. For example, in a mentoring initiative designed for Hispanic/Latina/o/x students, professionals served as role models for academic and career advancement (Sanchez et al., 2022). Through their engagement with adult professionals, youth in another program began to make connections between education, careers, and their economic potential (Cayleff et al., 2011). This allowed them to construct a coherent plan that bridged their current activities to a greater sense of purpose and goal-orientation. An alumna of a college and career readiness program for low-income youth from the U.S. and other countries shared, “[My internship] definitely opened up my perspective in figuring out what I needed to get out of college… [This] drove what I would study and prioritize during college” (Detgen et al., 2021, p. 239). These experiences in organized activities helped youth develop an understanding of the pathways to careers, college, and goal achievement, while motivating them to take action to increase their likelihood of future success.

##### Youth Commitment to Paying-It-Forward

Participation in organized activities can foster a commitment to paying-it-forward, both within the program and the greater community context. For instance, some organized activities intentionally built in opportunities for the youth to become interns or mentors to incoming cohorts (Ching et al., 2016; Cox, 2017). Regardless of whether these formal opportunities existed, appreciation for the support received from staff inspired many young people to serve as a positive influence for other youth like them (e.g., Kniess et al., 2020; Miller, 2011). A youth-mentor in an academic support program shared with his peers: “Keep in mind that whatever you guys do, the next cohort sees… What example are you setting, right? Ask yourselves that. And take it seriously” (Cox, 2017, p. 54). Taking this sense of reciprocity, a step further, programs designed for African American/Black and Hispanic/Latina/o youth that involved civic engagement facilitated the development of collective consciousness by providing opportunities for youth of color to have a say in community affairs (e.g., Ginwright, 2007; Sullivan & Larson, 2010). For example, one program that offered support for youth to lobby their local government institutions empowered youth of color to promote systemic changes that would benefit others like them (Sullivan & Larson, 2010). Thus, activities that provide opportunities for youth leadership foster high regard for reciprocity and encourage youth to leverage their own social capital in support of their community.

##### Youth Self-Efficacy in Reaching Goals

Participation in organized activities strengthened youths’ sense of self-efficacy in achieving their goals. Many organized activities supported youths’ self-efficacy in reaching life goals by teaching and encouraging young people’s growth in academic skills (Bempechat et al., 2014), relationship-building skills (Schwartz et al., 2016), and skills for enacting agency such as professional communication, time management, and leadership (Powell et al., 2017). Further, experience in professional settings helped youth develop confidence that they could thrive in these environments (Ching et al., 2016), while also providing opportunities for youth to feel accomplished (Dill & Ozer, 2019). As youth developed greater self-efficacy, they were more likely to frame their personal characteristics as strengths (Harris & Kiyama, 2015) and demonstrated more comfort engaging in potentially daunting tasks (Ching et al., 2016; Miller, 2011).

##### Youth Relationship-Building Skills

Youth in organized activities noted how the programs they were involved in helped them develop relationship-building skills, communication skills, social skills, and confidence in interacting with adults and professionals (e.g., Kniess et al., 2020; Sullivan & Larson, 2010). Other organized activities emphasized the importance of building networks (e.g., Habig et al., 2021; Murillo et al., 2017), providing more explicit opportunities to build networking skills including social networking events (e.g., Museus, 2010), and giving youth specific strategies for maintaining relationships and building new connections (e.g., Schwartz et al., 2016). Organized activities supported and encouraged relationship-building skills among youth by providing opportunities for them to initiate and build relationships with many types of individuals. For example, “a key component of the ways in which targeted support programs create early connections is by offering students opportunities to connect and develop meaningful relationships with their college peers, administrators, counselors, and academic advisors…” (Museus, 2010, p. 26).

### Youth Social Capital Development

The proposed model shows that organized activities are well positioned to support youth in strengthening and leveraging their social capital by creating a relationally strong climate, which in turn, contributes to increased social capital opportunity and activation. Social capital opportunity refers to youth experiencing increased and more consistent developmental relationships with adults and peers within the organized activity. It is through these relationships that youth increase their access to valued resources (e.g., financial help, information, guidance) including new connections and relationships outside of organized activities that can be used as youth pursue life goals (i.e., social capital activation).

#### Increased Social Capital Opportunities

Increased social capital opportunities were promoted by organized activities that facilitated developmental relationships between staff and youth, which in turn increased access to valuable resources and connections that were not previously available.

##### Developmental Relationships with Staff and Peers

The relationally strong climate fostered by staff and youth mindsets and skills resulted in youth experiencing stronger relationships with program peers and staff, which contributed to a web of support. For example, one youth described how through their program they have a lot more people to challenge and support them, “… I don’t think I would have as much passion as I do because I know so many people… know that I can do it and like, I will make them proud” (Lane & Id-Deen, 2020, p. 18). Similarly, youth in a school-based club for girls described how they now talk to peers that they would not have previously, but the program “opens up room for us to get to know each other better” (Cayleff et al., 2011, p. 34). A study of an entrepreneur program similarly noted how every young person interviewed emphasized the important role that relationships with multiple staff members and mentors played in their personal growth and in expanding their network (Miller, 2011). Many of these relationships extended beyond the life of the program and served as important sources of support later on. For example, a college and career readiness program described how “lifelong friendships” among peers in the program were formed and served as a support group (Detgen et al., 2021, p. 242). Youth in organized activities consistently emphasized the web of developmental relationships with staff and program peers that they built through their participation in their respective programs.

##### Increased Access to Resources

It was through the developmental relationships formed within organized activities that youth gained increased access to a variety of resources. Some of the organized activities provided financial assistance to young people (e.g., Dill & Ozer, 2019; Erbstein, 2013), whereas others learned about financial assistance and other funding opportunities through staff (e.g., Garcia et al., 2021; Sullivan & Larson, 2010). Many of the organized activities were designed to give young people opportunities to explore potential postsecondary opportunities or to provide access to these opportunities through internships, work-based learning, and near-peer mentoring (e.g., Ching et al., 2016; Koch et al., 2019). Organized activities also gave youth opportunities to practice and learn new professional and interpersonal skills that would be valuable for their future goals including communication, time management, organization, study skills, and social and networking skills (e.g., Bempechat et al., 2014; Hernandez-Gantes et al., 2018). Through relationships with staff and peers, young people also often gathered advice and information on navigating the education and employment system. This often included understanding college jargon, assistance with filling out paperwork, and connecting with mentors for advice on applying to jobs and/or greater education. Finally, organized activities often connected youth to others outside of the organization who were useful for reaching life goals. This could include connecting young people with individuals within the community, professional mentors, professors and educators, and potential employers, among others.

#### Increased Social Capital Activation

Through the mindsets and skills youth developed in organized activities, they learned how to capitalize on the social capital opportunities afforded by these activities in support of pursuing their interests and postsecondary and/or life goals, often by taking the initiative to engage or seek help, even outside of their involvement in the activity. For example, one program highlighted how a young person “shared that she sustained her relationship with Ashley [staff member] beyond high school by seeking out help during the summer before her first semester in college” (Ramirez, 2021, p. 1073). Several other organized activities described how youth secure their own mentors, seek help from educators, intentionally build new relationships and professional connections, and learn skills to advocate for oneself (e.g., Ching et al., 2016; Schwartz et al., 2018; Tichavakunda, 2019). It is through this activation process that young people recognized that they can “leverage these resources [gained through participation in an organized activity] for long-term success” (Dill & Ozer, 2019; p. 1622).

## Discussion

Little is known about the role and malleable features of organized activities that can be leveraged to support youth social capital development. Therefore, the current study provided a meta-synthesis of extant qualitative research that illuminates the developmental pathway through which social capital development occurs in organized activities. The synthesis revealed a tangible set of malleable organized activity features that act as levers for social capital promotion. These included elements at the organization level (e.g., organizational partnerships), strategies that foster a relationally strong climate (e.g., creating a safe space), and key skills and mindsets that both youth and staff possess that contribute to this climate. As illustrated in the proposed model (see Fig. 2), all of these elements synergistically work together to cultivate opportunities for youth to build relationships within and outside the organized activity that provide access to resources which youth, in turn, can activate as they work towards their education and career goals. In addition to activity features that support social capital development, youth engage in behaviors (e.g., actively and strategically reaching out to their web of support, securing resources needed to reach specific goals) that catalyze their ability to reach these life goals. This finding is consistent with past research that suggests the use of social capital requires a level of agency or mobilization on the part of the young person (Boat, et al., 2022a).

The meta-synthesis showcased the important role and impact of social capital at the organization level. This finding is consistent with social capital theory, which highlights social capital beyond individuals to include social capital at organizational and community levels (Payne et al., 2011). For example, past research has shown how institutions in low-income neighborhoods can serve as resource brokers and provide bridging and linking forms of social capital through their ties to other organizations (Small, 2006). In addition to organizational partnerships, findings highlight several supporting structures that were helpful for cultivating a climate that prioritized youth social capital development. These included inputs like a shared commitment to the organized activity’s mission and/or values, staff retention, and hiring staff that share similar racial/ethnic identities and/or life experiences as youth participating in the program. It is also possible that there are other valuable structures that went unmentioned in the reviewed articles. Past research in the positive youth development field has long shown that family engagement, staff professional development, staff recognition, and resource allocation may also be important supporting structures for creating a strong organizational climate (National Research Council, 2002).

The synthesis identified several elements that are characteristic of a relationally strong climate, including creating a safe and culturally responsive space where youth have a strong sense of community and belonging and are provided opportunities to explore their sparks and deep, personal interests. Furthermore, the themes showed that both staff and youth have specific skills and mindsets that likely nurture this climate. For staff, this involves believing in youth as assets and potential and exhibiting the skills needed to build positive relationships with youth while also brokering resources on their behalf. For youth, the studies highlighted how participation in organized activities strengthened relationship-building skills and mindsets such as future orientation, self-efficacy, and commitment to paying-it-forward to others. These youth mindsets and skills are also likely to strengthen youth’s ability to actively mobilize relationships and resources as they work towards their life goals (i.e., increased social capital activation).

### Organized Activity Elements that May Be Particularly Important for Youth from Marginalized Communities

While this study aimed to synthesize the factors among organized activities that contribute to the social capital development of all youth, we identified factors that may be particularly important for youth from marginalized backgrounds. These features included building positive relationships with staff members and other roles models who share the same racial and ethnic identity as youth participants, providing culturally responsive programming and a safe space to discuss systematic barriers, and supporting youth with larger-purpose goals such as changing systems of inequity and paying-it-forward to others from similar backgrounds or communities.

Several organized activities noted how it was beneficial for youth of color in their programs to experience positive relationships with staff members with the same racial or ethnic identity. When youth see staff members and/or build connections with mentors who look like them, it can reinforce youth’s belief that they too can succeed in pathways similar to these role models (e.g., Richardson, 2012; Woodland et al., 2009), and may empower youth to embrace and explore their own racial or ethnic identity leading to a stronger sense of self and pride in their identity (Ginwright, 2007; Harris & Kiyama, 2015).

Staff members who share the same racial and ethnic identity as youth participants possess a common lived experience and may as a result have a deeper understanding of the cultural nuances and challenges that youth from that background may face, and therefore may be more likely to develop “culturally-relevant relationships’’ (Dill & Ozer, 2018). The synthesis highlighted several examples (e.g., culturally-relevant content, attending to language needs, family-program partnerships) of culturally-relevant relationships leading to staff being more attuned to the cultural and social needs of youth and the provision of more effective and culturally-relevant support (Wong, 2008; Woodland et al., 2009). Strategies for creating a culturally-responsive environment for youth varied across the organized activities with some providing explicit cultural competence training to staff and mentors (Ramirez, 2021; Sanchez et al., 2022) and others intentionally providing safe spaces for staff and program youth to talk about systemic barriers and experiences of discrimination (Cox, 2017). In some cases, organized activities also inspired and supported youth to challenge deficit-based narratives around their racial or ethnic identity and to engage in social change efforts. One leadership program designed for Black youth, for example, aimed to create a “collective Black youth identity” that equipped Black youth within the program to address social issues within their community (Ginwright, 2007).

The social capital literature traditionally has focused on the individual’s pursuit of educational and/or career goals (e.g., reviewed in Scales et al., 2020), and has for the most part given far less attention to what might be called larger-purpose social capital development goals beyond the individual youth’s achievements. Yet this synthesis has shown that social capital development, particularly for youth from marginalized communities, often involves two other integral, larger purpose “goals”: namely, helping youth change/dismantle systems of inequity, and helping youth pay-it-forward or give back to their communities and those who follow them.

Staff and mentors in the organized activities also helped instill these larger purpose goals. For example, a young person in a mentoring program described the impact it had on them that their mentor who came from a low-income family went on to college and then decided to come back to their community to help other youth (Murillo et al., 2017). Organized activities that recognize and embed these larger purpose goals into their programming may be increasing social capital development effectiveness both for individual youth and society (Case, 2017). Future research should continue to examine culturally-specific themes or elements of organized activities that may be particularly important for youth from a variety of racial and ethnic identities and social class backgrounds (see similar calls in Case, 2017; Travis & Leech, 2013). These findings will likely lead to higher-quality youth programming that better meets the needs of youth served.

### The Social Context of Organized Activities

It would be remiss to not acknowledge the social context in which organized activities and young people are embedded (see Fig. 2). Previous research shows that both participation in organized activities and access to social capital is not equitably distributed, especially among young people from marginalized communities (Carpiano, 2006; Vandell et al., 2015). For example, there are an estimated 25 million young people who are unable to access a high-quality afterschool program, a figure that is more pronounced among youth of color and young people from lower-income communities (Afterschool Alliance, 2020). The Afterschool Alliance’s America After 3PM survey (a nationally representative sample of over 31,000 parents/guardians) documents some of the most commonly reported barriers that prevent youth from accessing these types of programs, including program costs, availability in the community, and access to safe transportation to and from programs, all of which were disproportionately identified by Black and Hispanic/Latina/o/x parents and parents from lower-income communities (Afterschool Alliance, 2020). The same dynamics have been found to result in much lower youth sports program participation by youth of color and from lower-income communities (Aspen Institute, 2022), with inequitable participation, access, and mental health getting worse over the course of the COVID-19 pandemic (Aspen Institute, 2022; Prime et al., 2020).

While the social context can limit access to social capital promoting organized activities, it can also nurture and thwart social capital development at any point across the proposed model including access to high quality staff who truly believe in youth’s potential, access to the appropriate resources needed to reach one’s goals, and access to the skills needed to mobilize one’s web of support. Even when all the conditions appear to align, there are dynamics of power, privilege, and discrimination that may still prevent young people from reaching or even pursuing their education and/or career goals. Therefore, while organized activities are one promising avenue from supporting social capital development among youth, researchers and practitioners alike need to continue to explore ways to further support youth, and in particular youth from marginalized backgrounds, in leveraging and building social capital and additional assets within their families, schools, and communities, while working to change systems that hinder this development.

### Implications for Practice

The current synthesis has important implications for the resource management and program design of organized activities committed to intentionally promoting youth social capital. The proposed model underscores a number of malleable elements that organized activities can intentionally strengthen in order to more effectively support the youth they serve. Perhaps one of the most malleable and impactful levers for supporting youth social capital development is the youth-adult developmental relationships that are formed within organized activities.

The current review highlighted the many different roles that staff play within organized activities including as *institutional agents*. Staff’s status as authority figures in their organizations positions them to provide bridging and linking forms of social capital by serving as brokers to their organization’s network of relationships and resources (Stanton-Salazar, 2011). Staff’s ability to broker resources that youth actually desire is determined by the resources that are available within the organization’s network. Yet, not all staff have the mindsets and skills needed to effectively build strong developmental relationships and broker resources on young people’s behalf, especially across lines of difference (e.g., cultural, racial, and ethnic identity). In this sense, organized activities hold more power than individual staff do to create systems level change. Therefore, it is essential that organized activities hire and retain staff that can meet these needs of young people and/or provide the training needed to support staff in building these skills and mindsets.

The synthesis also highlights the importance of organizational partnerships. When organized activities partner with youth’s families and other community organizations, they enhance the capacity of their staff by expanding their network of resources (Stanton-Salazar, 2011). Through this expanded network, organized activities are better able to provide wraparound supports that meet the holistic needs of the youth (Syvertsen et al., 2021). Additionally, these partnerships can operate as coalitions in service of community change, particularly when focusing on the needs of marginalized populations (Small, 2006). As such, they can increase collective efficacy and action to redress the many systematic barriers and inequities that prevent young people from marginalized communities from reaching their life goals. Yet, it is important to note that the ability to participate in organized activities is also often inequitably distributed and influenced by economic status, race/ethnicity, gender and other social factors. By virtue of participating in a high-quality organized activity, young people may have greater opportunity to strengthen their social capital.

### Limitations and Future Directions

Several potential limitations should be considered in future research. Although our search strategy was comprehensive, articles written in languages other than English were not included. We also recognize the limitation of language or terminology in the usage of the term *social capital*. It is possible that articles examining organized activities that support young people’s social capital may use other terms beyond what was included in our search terms. The current meta-synthesis also did not limit studies to a particular type of organized activity. Rather, many different types of organized activities were included such as mentoring programs, afterschool programs, sports, and community-based organizations. Future research may consider examining whether there are elements that are particularly important in these distinct contexts for promoting young people’s social capital development (e.g., authentic learning opportunities; youth-initiated tasks; working towards a shared goal; see Boat et al., 2022). We have put forth a proposed model for how organized activities may support youth social capital development. Future research should further refine this model as new empirical findings emerge and begin to empirically test these proposed pathways and linkages.

## Conclusion

Despite an increasing interest and value placed on organized activities and how the social relationships fostered within them can support youth in reaching their future goals, relatively few studies have attempted to capture the core elements that are needed in order to not only promote positive youth development in general, but to fully support youth social capital development. The current review sought to address this by reviewing and synthesizing the related literature on organized activities and social capital. The thematic analysis identified seven themes relating to the development of young people’s social capital. These themes were used to create a proposed model for how organized activities may support youth social capital development. These findings suggest potential strategies that organized activities and staff may implement into their practice. Moreover, these findings serve as a call for action for organized activities to consider how they might increase collective efficacy and action to redress the systematic barriers and inequities that prevent many young people from reaching their full potential.
